# Working as a nurse in community health services during Covid-19: a qualitative study

**DOI:** 10.1186/s12912-022-01141-4

**Published:** 2022-12-16

**Authors:** Ellen Benestad Moi, Anne Valen Skisland, Berit Johannessen, Kristin Haraldstad, Gudrun Rohde, Sylvi Monika Flateland

**Affiliations:** Department of Health and Nursing Science, Faculty of Health and Sport Sciences, University of Agder, P.O.Box 422, 4604 Kristiansand, Norway

**Keywords:** Community health care, Nursing, Nursing management, Psychological well-being, Safety

## Abstract

**Background:**

During the Covid-19 pandemic, new roles, increased workload, lack of staffing and infection control equipment, unclear infection control guidelines and conflicting information have led to uncertainty and unpredictability for health workers. Although community home-care nurses have been exposed to a range of personal and professional stressors during the pandemic, few studies have focused on their experiences. The aim of this study was to explore how Norwegian home-care nurses experienced the first wave of the Covid-19 pandemic. This knowledge may contribute to preparations for meetings with patients in future pandemics, how management can support its employees and how to structure a successful organization.

**Methods:**

This study was a qualitative descriptive design comprising 12 semi-structured individual interviews with home-care nurses. A thematic analysis was carried out.

**Results:**

Four main themes and 11 subthemes were constructed. The results revealed challenges related both to the organization and to management, experiences of unclear information, lack of available equipment, redeployment of staff and increased workload. Furthermore, it was challenging to provide high qualitative care. The nurses missed collegial togetherness and had feelings of uncertainty with a great fear of infecting others. Positive consequences were feelings of being valued and a greater awareness of infection control.

**Conclusion:**

This study highlighted the importance of unambiguous information and clear delegation of responsibility, and that enough infection control equipment will likely minimize the fear of infecting each other. Being visible and admired for their work was important for the nurses’ psychological well-being. Nurses, nursing managers and policymakers in community health care can use these results to develop strategies for future pandemic planning.

## Background

When the Covid-19 pandemic spread worldwide in March 2020, it led both to uncertainties and to rapid changes in the work situation for health-care workers. How government authorities and national health services have managed the pandemic and the number of confirmed cases has varied in different countries [1, 2]. A brief report that compared the prevalence of Covid-19 and related mortality among nursing homes in 14 countries confirmed a wide variation in case numbers and deaths [3]. Norway has generally shown both low case numbers and low death rates compared with other countries [4–6].

Infection control guidelines for health personnel in general have been unclear and developed day by day. Ranney et al. [7] emphasized in the early phase of the pandemic (March 2020), that protective equipment was missing, and health professionals should save it for the people who really needed it. Simultaneously, the recommendation was to use a facemask and adopt a regime to reduce droplet infection. Conflicting information may have led to uncertainty and unpredictability among health-care professionals [8, 9].

Strict and rapidly successive constraints have been imposed by government authorities such as: keep a 1.5 m distance, stay at home, lockdowns, border closures, recommendations for use of face masks and handwash. Norwegians have great confidence in the government’s recommendations, and most people follow the recommendations, which are updated daily [6]. Norway also has a public health service where everyone has the right to free treatment [10]. One year into the pandemic most people over 60 years of age and health personnel have been vaccinated and have had their booster [6].

A Norwegian study of nursing homes and home-care nursing highlighted the managerial challenges of following infection control rules such as maintaining physical distance between personnel and patients, monitoring personnel who work in multiple locations and spot disinfection [10]. Halcomb et al. [9] described how more than half of respondents, nurses in home care, felt well-supported by their employer. Furthermore, a third of respondents perceived that care provided in their workplace was significantly or slightly worse than before the pandemic [9]. While and Clark [11] indicated that nurse managers could make a difference to meet home-carenurses’ increased workload and prevent burnout. They pointed out that nurse managers should be nurturing a supportive organization where the workload was managed participatively, and self-kindness was legitimate [11].

A Norwegian report of nurses’ experiences from the first phase of the Covid-19 pandemic from March to October 2020 [12] indicated that health services were better prepared in November 2020 than in March 2020. The pandemic has led to major redeployment of staff, and the burden on the remaining employees has increased. At the same time, Norwegian nurses had to change their working hours, increase their workload and manage new work tasks [12]. The specialist health service was generally better prepared for a pandemic than the municipalities [12]. Despite the specialist health service being better prepared, the experiences of community home-care nurses seemed to be very much akin to those of nurses in other settings [12]. Previous studies have shown that community home-care nurses have been exposed to a range of personal and professional stressors during the pandemic that have impacted their psychological well-being [8, 9, 13]. Important findings were the feeling of being valued and that their role was respected by both the community and their workplace. The psychological impacts consisted of anxiety about increased workload, lack of staffing, worries about transmitting Covid-19 to their family and patients, and conflict between duty and safety [8, 9, 13]. Self-care strategies and motivation included increased vigilance of infection control at home and at work, and attention to physical exercise and diet. Other important factors were adequate personal protective equipment, information, family support, concern for quality in nursing given to certain patient groups, reasonable staffing and bonus pay [8, 9, 13].

The majority of studies have focused on patients and frontline hospital staff and, to a lesser extent on nurses in home-care service. Although Norway has had relatively few cases of infection with Covid-19 compared with other countries [4], the experiences of home-care nurses in Norway will illuminate how nurses who visit patients in their own homes experience a pandemic. These experiences will contribute to the knowledge of how to prepare for future pandemics, both as health-care professionals in meetings with patients, how management can support its employees and how to structure a successful organization.

### Aim

The aim of this study was to explore the experiences of Norwegian home-care nurses in the first wave of the Covid-19 pandemic.

## Methods

### Design

This study adopted a qualitative design [14, 15], using individual semi-structured in-depth interviews. A guide was used as a checklist.

### Sample selection and context

This study was conducted in the Norwegian community health-care sector. Community-based health-care services are characterized by professional health-care teams, working in patients’ homes. The selection of participants was made using strategic sampling [15]. The local manager in one municipality in South Norway was contacted by email and asked if they could recruit RNs for an interview study. The inclusion criteria were RNs working a minimum of 50% of their work time in community health-care services during the pandemic, from March 2020 until the start point of the study. Twelve females RN fulfilled the inclusion criteria and accepted the invitation.

### Data collection

The participants received oral and written information about the study, and informed consent was obtained from all (nurses) before the interviews. All the participants in the study were informed that they could discontinue the participation at any time without stating the reason. Four of the authors (EBM, BJ, AVS and SF) carried out the interviews from January to March 2021. The participants were asked to reflect on their working situation during the Covid-19 pandemic, including questions such as: How is/was it to work as a nurse during the Covid-19 pandemic? What are the main differences between working as a nurse before and after the pandemic broke out? Probing questions were asked to gain further insight. The participants were individually interviewed, face to face or digitally. Six out of twelve interviews were conducted online, via Zoom. A web link was sent to the participants before the interview. After twelve interviews the collected data reached saturation.

### Analysis

The interviews were recorded and transcribed verbatim and were analysed in six steps according to Braun and Clarke’s [14] thematic analysis. The steps were as follows: (a) individual interviews were read several times by the authors searching for meanings and patterns in themes. Some of the patterns were discussed among the authors; (b) two of the authors (EBM, SF) coded the patterns, and the codes were cross-checked by all the other authors; (c) based on the initial codes, all the authors searched for common themes; (d) all the themes were reviewed; (e) the themes were named and finally; (f) the themes were reported based on evidence from the data. The participants did not comment the transcripts but were informed of the possibility of contacting the interviewers if they had new points they wished to convey or remove statements.

## Results

The selected sample is representative of this group characteristic according to gender and age distribution and experiences. None dropped out. The age of the participants ranged from 24 to 46 years (mean = 31.8 years), and they had worked from 2 to 20 years in the municipality (mean = 6.4 years) (Table 1).

**Table 1:** Sociodemographic characteristics of participants

Participants	Age	District	Number of years as a RN in the district
1	28	1	5
2	25	1	4
3	30	3	4
4	29	2	4
5	29	2	7
6	25	2	4
7	43	2	2
8	30	3	4
9	28	4	2
10	46	4	19
11	45	4	20
12	24	1	2

The interviews lasted from 21 to 55 min (mean = 38.36 min).

### Findings

The result describes how RN´s experience their daily work as challenging both on the organizational and individual level. The analyses resulted in four main themes and 11 subthemes (Table 2).

**Table 2:** Themes and subthemes identified in the study

Main Themes	Challenges related to organization and management	Challenges to provide high qualitative care	From uncertainty to safety	Positive consequences of the pandemic
**Subthemes**	• Unclear information and lack of available equipment • The importance of a supportive leader • Different perceptions of workload	• Convey safety behind a face mask • Absence of physical closeness in the workplace • Challenges in the exchange of patient information	• Feeling forgotten and lack of predictability • Fear of infecting others • Absence of collegial community	• Being valued and visible • Greater awareness of infection control

### Challenges related to organization and management

#### Unclear information and lack of available equipment

Nurses experienced a significant lack of information and explicit guidelines at the start of the pandemic. When the public guidelines emphasized the extensive benefits of using a face mask, the nurses were instructed not to use one. Consequently, some patients and next of kin purchased face masks for the nurses. Limited information from the employer led to an increase in communication between the different local offices. They experienced the same prevailing uncertainty among all the local offices in which they were employed. One nurse experienced it like this:

“… so, there was very much uncertainty in the group since some heard that some districts do this, and some districts do this and here we don’t do either. Why and what and who should decide how to deal with this? And what can we do here?” [Nurse 11].

The nurses kept up with the national news themselves and with the guidelines that were given at a national level. Significant uncertainty was prevalent both nationally and locally and the uncertainty was reinforced in their specific work situation.

“Heard on the radio on the way to work. The Assistant Minister for Public Health said that face masks should be used in home-care nursing, but we did not have any routines in place related to the use of face masks” [Nurse 10].

The district offices appointed staff without special infection control competency, to be resource persons for infection control. These nurses had to draw up new procedures and protocols that were in accordance with the Norwegian Institute of Public Health and the local guidelines related to the pandemic.

One challenge that was particularly prevalent at the start of the pandemic was the lack of equipment. One nurse told how they ordered huge amounts of infection control equipment and yet received only small numbers.

“We ordered 500 facemasks. We received 25. It was impossible to get a hold of … it was a real blow to us … glasses and visors were impossible to get a hold of. We had to go to Europris (a cheap store chain in Norway) and buy protective glasses used for fireworks. That was the level we were at!” [Nurse 7].

#### The importance of a supportive leader

As well as challenges associated with obtaining enough equipment, our participants experienced the importance of how their immediate superior and their district manager acted after the outbreak of the pandemic. Many had positive experiences with their manager regarding how they dealt with the challenges. One participant experienced it like this:

“They have been very available to us, and there for us. They have kept us well informed, they made sure we were given the necessary amount of input and training. We had training days on the internet where the focus was on infection control” [Nurse 4].

#### Different perceptions of workload

All the nurses experienced a change in their working days when the pandemic hit, but only a few experienced that it became laboursome. The increased workload was related to the time used in getting dressed in infection control equipment, but it happened rarely. Some nurses experienced that several patients were reduced after the pandemic hit, because.

“…in our zone eight to nine patients phoned us immediately and wanted us to “telephone-visit” them instead of visiting them at home” [Nurse 3].

One nurse expressed how satisfying it was to have the possibility to go to work when everything in the society was locked down [Nurse 9].

### Challenges to provide high qualitative care

#### Convey safety behind a face mask

After a while with updated guidelines, everyone wore a face mask. The participants showed creativity in providing safety and instilling trust with patients and next of kin. One participant said:

“I have noticed myself that when I smile now, I, like, squeeze my eyes a bit extra so you kind of get those smiling eyes. I don’t normally do that when I smile, but now I smile a bit more like this (shows grimaces with the eyes). It is supposed to be more explicit, that I have a smile under my face mask. Yes, so there is a lot more body language now” [Nurse 8].

Others reported how they became more aware of being verbal with patients during the pandemic. While some raised their voices to convey safely, others spent more time with patients. It was particularly challenging to ensure safety and gain trust in patients with different forms of dementia illnesses. One of the participants shared her experience in this way:

“I wait to put on the face mask until the user sees me because many are living with dementia and therefore don’t recognize me with a face mask. It is a good idea to let them see us first, and then put on the face mask” [Nurse 4].

#### Absence of physical closeness in the workplace

Communication and being together with the nurse were for some patients the only social interaction during the day. Nurses felt it was important for many of the patients and that the daily meeting between nurse and patient or next of kin was much appreciated. One participant reported the following:

“Patients say that now it would be good with a hug … it is bad when users say that they have not been hugged in a year. And there they sit alone in their house or apartment” [Nurse 4].

When patients asked for more closeness, the nurses experienced this to be difficult. One nurse shared a quote from one of the patients: “You don’t need to use a face mask with me, because I will be dying soon anyway” [Nurse 3].

All the nurses experienced that the limitations related to closeness to the patient and next of kin influenced the quality of the work they did. They experienced a desire among the patients that affected the nurses and made them feel they did not perform their work as they would have liked. One of them explained it in this way:

“I don’t know, yeah, it is difficult to explain, but you can say a lot of nice things with words, and you can show compassion with body language in other ways, but it is something, yeah, just to be able to hug someone and in a way show them with your whole body that yes, I feel with you in a way, yeah” [Nurse 4].

#### Challenges in the exchange of patient information

When opportunities to meet became less and the verbal communication flow between colleagues was almost completely absent, the nurses experienced that this influenced negatively on the quality of their work. Contact with the treating doctor that they were used to became much less frequent. One of the nurses expressed:

“Before the pandemic, when we met each other face-to-face, either sitting together eating lunch, having meetings together or only passing each other, it was easier to raise questions [about the patients] that occurred during the day [Nurse 5].

When the meetings took place digitally, they tried to avoid questions that they felt could have consequences for the quality of the work they did.

### From uncertainty to safety

#### Feeling forgotten and lack of predictability

When the pandemic hit Norway and the society locked down, the nurses experienced that several occupational groups underwent considerable changes in their working situation. While many were told to use their home office, their function with home-care services continued to be carried out as before. They told about an experience of a pandemic that to a minimal degree created limitations for performing their professional work. This was in contrast with, for example, what the case was for other health services. One of the participants described the experience like this:

“As soon as it was a lockdown, the world stopped up. The streets were empty around the place, but we were out. We were out and drove alone. And in the beginning, it was a very strange feeling, a bit uncanny Hmm, we felt we were a bit forgotten. What about us? How should we deal with this? There was talk about nursing homes and there was talk about hospitals, but home-care services were out everywhere, we were a bit forgotten” [Nurse 11].

Other communal health profession groups, for example psychiatric nurses in the municipality, were instructed to follow up patients using the telephone. This resulted in them contacting the district offices and transferring some of the assignments they had responsibility for to the nurses there. One of the participants explained:

“There was a psychiatric nurse who called us and said that now I have a home office, can you make sure to shop for that user?” [Nurse 10].

Other municipal occupational groups like occupational therapists were transferred to district offices to assist nurses in their daily work. This was often done against the nurses’ will.

#### Fear of infecting others

The nurses were also afraid of infecting the patients, their own family and infecting colleagues. One of the participants said:

**“**All of us were afraid of being the one that would start an outbreak among colleagues and the elderly. It was a source of huge fear among the staff“[Nurse 11].

#### Absence of collegial community

As well as the fear related to infecting others, the nurses shared how they missed being able to relate to other colleagues at work. One nurse explained it like this:

“I get a lot of joy from being with my colleagues … it is so nice to come in, sit down and have a good chat with my colleagues … now I feel like if you come in a bit later to your break … there are just five places to sit over there … so you must sit in a separate room because you can’t sit there. And then you feel a bit sad, and so … you don’t get to talk to someone, and so it is just oh …” [Nurse 1].

The participants said that they missed being able to give each other support and comfort each other when they had experienced situations that were challenging: “…miss being able to be near each other and give each other a hug if you experience something bad at work” [Nurse 1].

### Positive consequences of the pandemic

#### Being valued and visible

The nurses experienced that they were given positive attention from the people in the local community related to their work. This was especially prominent at the start of the pandemic as they had not experienced this type of positive attention from local people in relation to their work previously. One participant said the following:

“I remembered I noticed this particularly at the beginning …, if you met a passer-by on the street, like … on the way to someone you were going to, suddenly everyone was smiling at you because they saw you were a nurse … no-one did that before like. Then you were just a person that walked past them, but now suddenly you were very much seen by complete strangers …” [Nurse 8].

The nurses also experienced increased attention at a national level, from politicians and people with central positions in society. The home-care nurses explained how much this increased attention meant to them. They believed they came closer as a professional group. They all had delivered good service to patients and next of kin, despite experiencing that the work was challenging at times. One of the participants experienced it like this:

“I believe that this has resulted in us being more connected across society because we are like together in this, and will hang in there and persevere together, and make this work somehow” [Nurse 8].

#### Greater awareness of infection control

The participants had gained a much better understanding of infection control than previously, in addition to the increased attention from the wider society. Infection control routines were drawn up in all the districts and participants felt the routines to be clear. They had gained knowledge and experience that gave them increased confidence to confront new pandemics.

“We have all achieved a better understanding of hygiene, that we need to be far more careful in following the rules. This we have really learned!” [Nurse 3].

## Discussion

The purpose of this study was to explore how Norwegian home-care nurses experienced their work during the first wave of the Covid-19 pandemic. The main results showed challenges related to organization and management, unclear information and lack of available equipment, redeployment of staff and increased workload. Furthermore, it was challenging to provide high qualitative care; they missed collegial togetherness and had a feeling of uncertainty with a great fear of infecting others. Positive consequences were based on being valued and visible, and greater awareness of infection control.

The nurses became insecure and experienced unpredictability concerning equipment and what rules and protocols they should follow. Experiencing such daily dilemmas was difficult, but they still faced the challenges. Other studies confirm that conflicting information during covid led to uncertainty and unpredictability [7–9, 16]. The informants in our study experienced challenges in developing new evidence-based procedures and protocols because the evidence and government protocols changed rapidly. Halcomb et al. [9] describe changes in nurses’ work by increasing the focus on developing triage tools and educating staff. However, the study does not describe the evidence for, or quality of, the new infection control protocols.

Our results further show that while conflicting information from the government was a challenge for management, the participants experienced frequent information, positive feedback and support from their management and employer during the pandemic. In contrast, just 54.8% in an Australian study [9] and 52% in a Norwegian study [12] felt well-supported by their employer. This may indicate that information and support from the management was experienced as adequate, and there was no need for more information at that time.

The redeployment of staff resulted in care for unfamiliar patients or laboursome work. Simultaneously, the participants felt responsible for helping patients and colleagues during the pandemic. Studies confirm that nurses had to change roles and work tasks [9, 17], and that nurses were motivated by ethical duty to care for patients despite the risk of infection [8].

The ability and willingness to adjust their workload and tasks in a crisis say nothing about how long the nurses can manage to tolerate such situations without having psychosocial challenges. Surprisingly, none of the nurses in our study expressed burnout symptoms such as sleep problems, despair, mental breakdowns, anxiety attacks or negativity as studies in other countries have described [8, 9, 11, 13, 17, 18]. We may assume that this is related to strict constraints by the government, the low frequency of infected patients in Norway [3, 6], and therefore the exposure of factors related to experience of lack of control and work-related stress did not affect our participants as much as expected. Studies demonstrate differences in work-related stress, distress and perceived safety, trust, and efficacy between varied healthcare workers during Covid-19 [18, 19]. Nevertheless, work-related stress and burnout arising from the Covid-19 pandemic is a crucial area of focus [8, 11, 13, 17–19]. Nursing management must have enough knowledge about their employees to identify who may need more support, and to serve as advocates to protect the physical and mental well-being of nurses [8, 9, 13].

The participants in our study were afraid of infecting patients, colleagues and family and had little social contact with family or colleagues. This fear is also highlighted in other national and international studies [9, 11, 13, 17]. All the nurses expressed that the pandemic reduced their quality of work, especially in the relation to end-of-life care and communication with patients with dementia. They missed the opportunity to meet physically and exchange information about patients. Other studies confirm the reduced quality of care provided [9, 17], while one Spanish study discusses the role of telehealth in home care [17]. The pandemic has raised questions about more use of telemedicine. Video consultations can replace nonverbal facial expressions but cannot substitute for face-to-face meetings, even with the use of facemasks.

The participating nurses describe feelings of being valued by having an important profession, both by the community, but also by management, employers and the government. They felt proud and said that being valued by others meant a lot to them. A study from Ashley et al. [13] also highlights the importance of professional and public support and acknowledgement of nurses’ roles during the pandemic, which may positively influence feelings of being valued. Being seen is important as a human being, and can help prevent stress, especially in a pandemic [11, 13].

### Strengths and limitations

This study was conducted during the Covid-19 pandemic and because of the pandemic situation it was difficult to recruit nurses. It is a limitation that all the participants were recruited from the same area, and all were female. Some of the interviews were conducted online via Zoom. Having a conversation online can be challenging, and it is difficult to register eye contact and body language, which may limit the understanding of the participants’ words.

On the other hand, to strengthen the study’s credibility we have emphasized transparency in our choice of methodology and described the research process in detail. It is also a strength that all the included nurses had many years of experience with community health-care nursing, and they talked freely about their situation.

The study’s results cannot be generalized, but they are especially relevant to the health service in general, and for nurses and nursing managers working in community health care.

## Conclusion

The purpose of this study was to explore how Norwegian home-care nurses experienced the first wave of the Covid-19 pandemic. The nurses experienced different challenges connected to information and delegation of responsibility. It was also important to have enough equipment and specific procedures and plans for reducing the spread of infection. As time went by, the uncertainty of the situation gradually decreased. Working during the pandemic made the health workers more visible to the Norwegian population who admired them for their effort during a very difficult time. Another positive consequence was that the nurses gained more knowledge about infection control. The pandemic seemed to be a wake-up call when it comes to having good contingency plans. Considering nurses’ experiences is valuable for future pandemic planning. Our findings may inform nurses, managers and policymakers in community health care. We recommend that future studies elaborate on the experiences of these results and focus on both nurses’ and nurse managers’ experiences after the pandemic.

## Data Availability

All data generated or analysed during this study are included in this published article

## Declarations

The nurses received oral and written information about the study, and informed consent was obtained from all participants. The study was conducted in accordance with the Consolidated Criteria for Reporting Qualitative Research (COREQ) checklist [22].
